# Investigating N-3 Fatty Acids to prevent Neonatal Tobacco-related outcomeS (INFANTS): study protocol for a double-blind, randomized, placebo-controlled parallel clinical trial of n-3 polyunsaturated fatty acids in pregnant smokers

**DOI:** 10.1186/s13063-021-05865-7

**Published:** 2021-12-14

**Authors:** Harvey J. Murff, Robert A. Greevy, Reesha S. Sanghani, Katherine E. Hartmann, Tina V. Hartert, Cornelia R. Graves, Scott S. Lee, Hilary A. Tindle

**Affiliations:** 1 Division of General Internal Medicine and Public Health, Vanderbilt University Medical Center, 2525 West End Avenue, Suite 450, Nashville, TN 37232 USA; 2 Geriatric Research, Education and Clinical Center, Tennessee Valley Healthcare System, Nashville, TN USA; 3 Department of Biostatistics, Vanderbilt University Medical Center, Nashville, USA; 4 Department of Obstetrics and Gynecology, Vanderbilt University Medical Center, Nashville, USA; 5 Division of Allergy, Pulmonary and Critical Care Medicine, Vanderbilt University Medical Center, Nashville, USA; 6 Tennessee Maternal Fetal Medicine, Nashville, TN USA

**Keywords:** Smoking cessation, Pregnancy, n-3 long chain poly unsaturated fatty acids (LCPUFA), Placebo-controlled, Pre-term birth, Red blood cell Membrane phospholipids

## Abstract

**Background:**

Tobacco use during pregnancy is the most important modifiable risk factor associated with adverse pregnancy outcomes, increasing the risk of preterm birth, intrauterine growth restriction and sudden infant death syndrome. Fewer than half of pregnant smokers can quit on their own. Identifying safe and effective therapies to prevent tobacco-related adverse pregnancy outcomes and/or increase smoking cessation in pregnant women would have a substantial public health impact. Cigarette smoking is associated with a relative deficiency in circulating n-3 long-chain polyunsaturated fatty acid (n-3 LCPUFA) levels. A recent analysis found that smokers taking n-3 LCPUFAs during pregnancy had a reduction in preterm labor risk when compared to non-smokers. Studies have shown that supplemental n-3 LCPUFAs may also reduce nicotine cravings and daily cigarette use. Thus, smokers may benefit from supplemental n-3 LCPUFAs by lowering the risk of preterm labor and/or increased smoking cessation. To address important remaining knowledge gaps, we propose the Investigating N-3 Fatty Acids to prevent Neonatal Tobacco related outcomeS (INFANTS).

**Methods:**

The INFANTS study is a multicenter, randomized, double-blind, placebo-controlled study that will randomize 400 pregnant smokers to either supplemental n-3 LCPUFAs or placebo. Participants will be enrolled between 12 and 24 weeks’ gestation and followed until 6 weeks after delivery. We will recruit from clinical centers throughout Middle Tennessee. We will assess smoking behavior after 12 weeks of supplementation using self-report and validated biomarkers of tobacco exposure. We will measure response to supplementation using biological markers of n-3 LCPUFA status. Our primary endpoint will be preterm labor as reflected by gestational age at delivery. Our secondary endpoint will be change from baseline in cigarettes per day at 12 weeks.

**Discussion:**

This study tests the hypothesis that smoking-induced n-3 LCPUFA deficiencies contribute to tobacco-related adverse pregnancy outcomes and that supplementation of n-3 LCPUFAs in pregnant smokers may prevent these complications. If our study demonstrates that supplemental n-3 LCPUFAs are effective at reducing the risk of tobacco-related adverse neonatal outcomes and/or reducing tobacco use during pregnancy, our results could have an immediate and major impact on pregnancy care and neonatal outcomes.

**Trial registration:**

ClinicalTrials.gov NCT04417595. Registered on April 21, 2020

## Administrative information

NOTE: the numbers in curly brackets in this protocol refer to SPRINT checklist item numbers. The order of the items has been modified to group similar items.

**Table:** 

Title {1}	Investigating N-3 Fatty Acids to prevent Neonatal Tobacco-related outcomeS (INFANTS): Study protocol for a double-blind, randomized, placebo-controlled parallel clinical trial of n-3 polyunsaturated fatty acids in pregnant smokers
Trial registration {2a and 2b}.	NCT #: NCT04417595 All items from the WHO Trial Registration Data Set are accessible on ClinicalTrials.gov
Protocol version [1]	Protocol Version 1, April 21,2020
Funding {4}	*Eunice Kennedy Shriver* National Institute of Child Health and Human Development, National Institutes of Health HD098719
Author details {5a}	Harvey J. Murff, MD, MPH^1, 2^, Robert A. Greevy, PhD^3^, Reesha S Sanghani MD, MPH^4^, Katherine E. Hartmann, MD, PhD^4^, Tina V. Hartert, MD, MPH^5^, Cornelia R. Graves, MD^6^, Scott Lee, MD, PhD^1^, Hilary A. Tindle, MD, MPH^1, 2^ ^1^Division of General Internal Medicine and Public Health, Vanderbilt University Medical Center, Nashville, TN; ^2^Geriatric Research, Education and Clinical Center, Tennessee Valley Healthcare System, Nashville, TN; ^3^Department of Biostatistics, Vanderbilt University Medical Center; ^4^Department of Obstetrics and Gynecology, Vanderbilt University Medical Center; ^5^Division of Allergy, Pulmonary and Critical Care Medicine, Vanderbilt University Medical Center; and ^6^Tennessee Maternal Fetal Medicine, Nashville, TN
Name and contact information for the trial sponsor {5b}	Harvey J. Murff, MD, MPH 2525 West End Avenue, Suite 450 Nashville, TN 37232, USA Tel: +1 (615) 936-8319 Email: harvey.j.murff@vumc.org
Role of sponsor {5c}	The sponsors had no role in the writing of this report and the decision to submit the report for publication.

## Introduction

### Background and rationale {6a}

Smoking is the most important modifiable risk factor for adverse pregnancy outcomes [2–5]. Approximately 11% of American women report smoking during pregnancy, with higher rates in younger women and those with lower educational levels, and women residing in the Southeastern United States [6, 7]. Rates of smoking at any time during pregnancy vary widely by state, from less than 5% to more than 25% [8]. Based on the 2010 Pregnancy Risk Assessment Monitoring System (PRAMS), in Tennessee, over 34% of women reported smoking during the 3 months before pregnancy and 22% reporting smoking in the last 3 months of pregnancy [7]. Up to 8% of preterm-related births, 19% of term infants with intrauterine growth restriction, and 34% of sudden infant death syndrome deaths are attributed to prenatal smoking [7]. Women who smoke have a more than 2-fold increase in the risk of preterm birth and low-birthweight neonates. Just as with smoking rates, preterm birth rates in states across the mid-South are also above the national average (e.g., 11% in Tennessee versus 9.6% nationally) [9–13]. The immediate and long-term care of preterm infants requires substantial utilization of medical resources with costs associated with preterm birth in the USA estimated at $26.2 billion in 2005 [14].

Rates of smoking cessation during pregnancy range from 25 to 75%, with risk factors for persistent smoking including lower educational status, higher number of cigarettes smoked per day, and coexisting psychiatric conditions [6, 7, 15]. Only 35% of pregnant smokers in Tennessee are successful at stopping smoking during their pregnancy, a proportion which is the second-lowest in the USA [7]. Promoting smoking cessation in pregnancy is challenging due to the limitations of available pharmacotherapy for pregnant and breastfeeding women for whom FDA-approved smoking cessation medications are generally not recommended [16–19]. Intensive behavior therapy alone in pregnant smokers produces quit rates as high as 35% by late pregnancy; however, most women continue to smoke despite counseling [20]. The identification of a safe and effective adjuvant therapy that reduces tobacco-related adverse pregnancy events and promotes smoking cessation in pregnant women would have a powerful clinical impact on maternal-fetal health outcomes.

Red blood cell (RBC) phospholipid membrane concentrations of n-3 long chain polyunsaturated fatty acids (LCPUFAs) are significantly reduced in smokers compared to non-smokers [21–25] We hypothesize that smoking-induced n-3 LCPUFA deficiencies may be an important mechanism contributing to tobacco-related adverse pregnancy outcomes and that n-3 LCPUFA supplementation specifically targeting pregnant smokers may reduce these complications. Support for this hypothesis comes from multiple observational studies which have reported lower levels of circulating n-3 LCPUFAs to be associated with adverse pregnancy outcomes, including preterm birth, intrauterine growth restriction, and preeclampsia, as well as higher risks for asthma in offspring [26–29]. Randomized controlled trials (RCT) of n-3 LCPUFA supplementation in pregnant women have had mixed results on preeclampsia risk and intrauterine growth restriction; however, recent meta-analyses have suggested a beneficial effect of supplemental n-3 PUFAs on preterm labor risk [30, 31]. To date, no RCT of n-3 LCPUFA supplementation for adverse pregnancy outcomes has prospectively stratified by smoking status, and only one RCT has done so post hoc [32]. The post hoc analysis revealed that among 136 smokers, supplemental n-3 LCPUFAs (2 grams/day) produced a lower risk of spontaneous preterm delivery (RR 0.56, 95% CI 0.36, 0.87), while no effect was seen among the 715 non-smokers (RR 1.04, 95% CI 0.84, 1.29, *P* value for interaction = 0.013) [32]. This analysis suggests that n-3 LCPUFA supplementation may protect against recurrent spontaneous preterm delivery and low birthweight. The large effect size observed provides a strong scientific premise to study supplemental n-3 LCPUFAs in pregnant smokers.

In addition to the potential beneficial impacts of n-3 LCPUFA supplementation in smokers on preterm labor risk, emerging evidence supports the potential role of n-3 LCPUFAs in directly modifying smoking behavior [33–36]. Indeed, two small, double-blind, randomized controlled trials have found that supplementation with n-3 LCPUFA reduces signs of nicotine dependence [37, 38]. Thus, the potential of offering a safe therapy to enhance smoking cessation efforts additionally supports the need for rigorous clinical trials of n-3 LCPUFAs in pregnant smokers.

### Objectives {7}

Based on the above preliminary studies, we hypothesize that smoking-induced n-3 LCPUFA deficiencies may be an important mechanism contributing to tobacco-related adverse pregnancy outcomes and that n-3 LCPUFA supplementation specifically targeting pregnant smokers may reduce these complications. In addition, we hypothesize that some of the benefit of n-3 LCPUFA supplementation in pregnant smokers is mediated through changes in smoking behavior and will test this hypothesis as a secondary aim of our proposed clinical trial.

### Trial design {8}

INFANTS is a placebo-controlled randomized parallel-arm trial of 400 pregnant smokers in the middle Tennessee area to test the effects of n-3 LCPUFA supplementation during pregnancy on the rate of preterm birth and smoking-related outcomes. Both study participants and study investigators and staff are blinded to treatment allocation

## Methods: participants, interventions, and outcomes

### Study setting {9}

Recruitment will take place at eight obstetrical clinics across the Middle Tennessee region. Clinical recruitment sites will be affiliated with Vanderbilt University Medical Center (VUMC) and Tennessee Maternal Fetal Medicine.

### Eligibility criteria {10}

In order to meet inclusion criteria for the study, the participant must be ≥ 16 or ≤ 40 years of age at the time of enrollment. She must report a current daily cigarette use of ≥ 1 cigarettes per day and report smoking 5 or more cigarettes per day prior to pregnancy. Daily cigarette use will be confirmed by an exhaled carbon monoxide (CO) reading of at least 4 ppm. The participant must be between 10 and 24 weeks’ gestation as estimated from the last menstrual period and adjusted for the first-trimester ultrasound at the time of enrollment.

Exclusion criteria include an allergy to fish, including fish oil supplements, and current use of fish oil supplements with unwillingness to stop over the course of the study. The participant cannot be actively using substances other than smoking, although they can be undergoing supervised buprenorphine therapy for opioid use disorder. The participant must be able to give consent or, in the case of minors, assent. Potential participants will be excluded if they have unstable pregnancy-related medical problems such as pre-eclampsia, known fetal or placental abnormality such as previa, accreta, increta, percreta, or multiple gestation. Potential participants will also be excluded if they have a history of hypertension as defined by chronic hypertension diagnosed prior to pregnancy, a seizure disorder, clotting disorder, or White’s classification D or higher diabetes. A planned cerclage or plans to move out of the area within the next 9 months are also criteria for exclusion.

### Who will take informed consent? {26a}

Potential participants who have been deemed preliminarily eligible will be approached in-person by study staff within the clinic before or after their normally scheduled visit. Once study staff has determined eligibility and thoroughly explained the details of the study, the potential participant will have the opportunity to ask questions, and, if willing, they will sign the informed consent or assent document. These activities will occur after the participants clinical visit and as part of study visit 1.

### Additional consent provisions for collection and use of participant data and biological specimens {26b}

All participants will be asked to sign a separate consent/assent for any ancillary studies in which they wish to participate. This will include a consent for the collection of additional blood for future genetic research.

## Interventions

### Explanation for the choice of comparators {6b}

We will use oleic acid (olive oil) as our placebo. The reason for the use of oleic acid is several-fold. Oleic acid capsules have a similar texture, size, color, and consistency to n-3 LCPUFA capsules and were used as the placebo in our preceding pilot work.

### Intervention description {11a}

Participants allocated to n-3 LCPUFA supplementation will be instructed to take four 1000 mg n-3 LCPUFA capsules (Metagenics™) daily. Participants will be instructed to take study medications with meals as concomitant consumption of n-3 LCPUFA supplements with meals increases tissue bioavailability [39–41].

The intervention period will have two phases. Phase 1 will be 12 weeks in duration, beginning at randomization (clinic visit 1) and ending at the in-person clinic visit 2. This phase will be used to determine impact on smoking behavior and compliance. Phase 2 will be the period from clinic visit 2 to 37 weeks gestation or delivery, whichever comes first. Medication dispensing will occur at clinic visits 1 and 2.

### Criteria for discontinuing or modifying allocated interventions {11b}

Fish oil capsules are generally well-tolerated, with minor gastrointestinal symptoms being the most common side effects. Participants reporting minor gastrointestinal symptoms (≤ grade 2) will be assessed to ensure they are taking the medication as suggested. Those who wish to remain on trial will be instructed to reduce their study medication from 4 capsules daily to 3 capsules daily. If symptoms persist or worsen over the next 3 days, they will be instructed to reduce their daily dose from 3 capsules per day to 2 capsules per day. If symptoms persist or worsen over the next 3 days, participants will be instructed to reduce their daily dose from 2 capsules per day to 1 capsule per day or, if necessary, 1 capsule every other day. If symptoms persist or worsen over the next 3 days, they will be instructed to discontinue the study medication.

### Strategies to improve adherence to interventions {11c}

Study participants will be provided with a pill diary upon study enrollment. Participants will be expected to track their medication adherence every day throughout the duration of the study. Between enrollment and 37 weeks’ gestation, subjects will receive text messaging reminders to encourage medication compliance.

### Relevant concomitant care permitted or prohibited during the trial {11d}

We will ask that the participant not take any fish oil supplements other than what has been provided by the study throughout the duration of their involvement in the study.

### Provisions for post-trial care {30}

There will be no provisions for ancillary or post-trial care for study participants.

## Outcomes {12}

### Preterm labor

Preterm labor is defined by gestational age at delivery, which will be abstracted from medical records by study staff. Gestational age at randomization will be determined according to a previously described algorithm on the basis of the last menstrual period and earliest ultrasound examination and will not be revised after randomization [42]. Gestational age will be estimated from the last menstrual period (LMP) and adjusted for the first-trimester ultrasound. If a self-reported LMP is greater than 7 days from the calculated ultrasound LMP, the ultrasound will be used to assign gestational age.

### Cigarettes per day

We will determine the percentage change from baseline in cigarettes per day (CPD) at 12 weeks. The outcome will be determined based on participant self-report to responses at the clinic visit 2 follow-ups. We will also determine change from baseline in end-expired CO and serum cotinine levels.

### Neonatal and maternal secondary outcomes

Neonatal secondary endpoint measures include the following: [1] fetal death and stillbirth, [2] individualized birthweight Z-score (adjusted for gestational age and maternal weight), [3] Apgar score, [4] intraventricular hemorrhage, [5] neonatal enterocolitis, [6] congenital abnormality, and [7] neonatal respiratory distress. Maternal endpoint measures include the following: [1] maternal mortality, [2] mode of delivery, and [3] hypertension in pregnancy. All neonatal and maternal endpoint outcomes will be collected by medical chart review after delivery.

### Smoking abstinence and smoking dependence secondary outcomes

Point prevalence abstinence from smoking at 12 weeks will be based on self-reported smoking cessation and biochemically confirmed by end-expired carbon monoxide and blood cotinine levels. Changes in nicotine dependence will be based on changes from baseline in the Fagerström Test for Nicotine Dependence [43].

## Participant timeline {13}

**Fig. 1:**
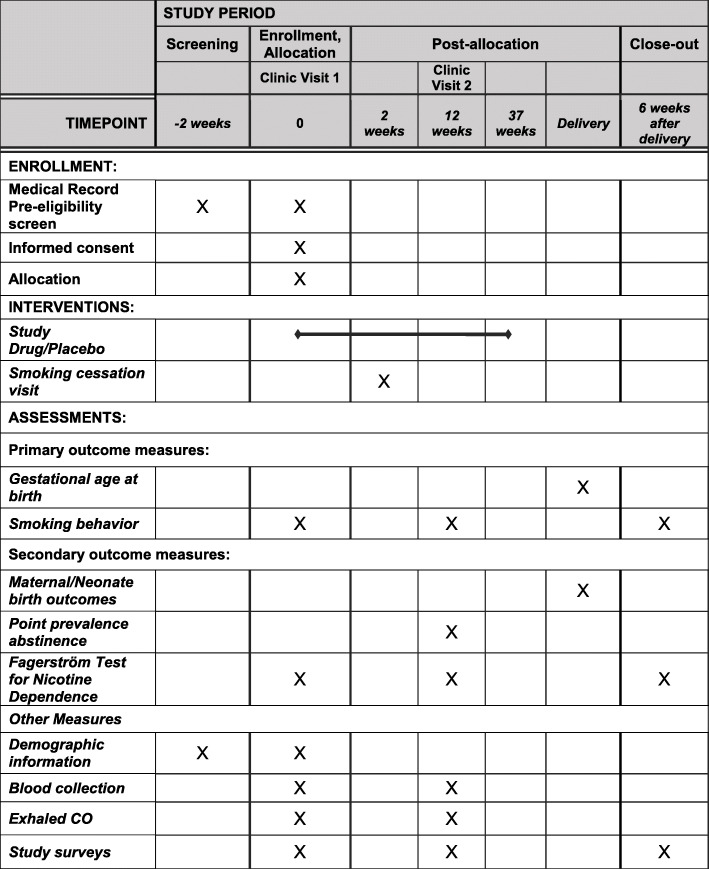
Organization of the INFANTS study

The study schedule of events is included in Fig. 1.

## Sample size {14}

We will recruit 500 subjects to ensure at least 400 observed outcomes, allowing for a 20% loss to follow-up rate.

## Recruitment {15}

Prior to recruitment, potential participants will be screened by study staff in order to establish potential eligibility. We will review clinic lists at all recruitment sites for upcoming visits. Tobacco use data is routinely collected by obstetrical nurses at study sites. Study staff will review clinic schedules on a daily basis to identify potential participants to approach after clinical visits. In addition, the PI’s will address providers and nurses at all clinical sites, introducing them to the study and distributing advertisement and contact information. Investigators will attend staffing meetings to introduce the study and take questions. Study staff will physically be located at clinics to attempt to identify potential participants who are new to the healthcare system.

Fliers and posters will be disseminated in clinical rooms, waiting rooms, and clinic bathrooms which will briefly describe the study and give appropriate contact information for potential participants to contact.

## Assignment of interventions: allocation

### Sequence generation {16a}

After providing informed consent at clinic visit 1, participants will be randomized based on permuted blocks stratified by smoking amount (< 10 cigarettes per day or ≥ 10 cigarettes per day), history of preterm labor, and presence of supervised buprenorphine use. The randomization schema was developed by the study statistician and is maintained by the Investigational Drug Service (IDS).

### Concealment mechanism {16b}

The IDS will assign subjects and dispense either active agent or placebo. Only IDS pharmacy staff will have access to the randomization schema.

### Implementation {16c}

Patient treatment assignments will be managed through a central randomization system by IDS. Aside from regulatory safety reporting requirements, IDS will not provide the study staff with any information that could potentially un-blind the therapy assignment of any enrolled patient. IDS will also be responsible for the storage, preparation, and labeling of drugs.

## Assignment of interventions: blinding

### Who will be blinded {17a}

All individuals participating in and conducting the clinical study will be blinded to patient therapy assignments until patient enrollment in the study has ceased, all enrolled patients have completed the study, and final data lock has occurred. This includes all study staff, clinic personnel, laboratory staff, study biostatisticians, and the participants.

### Procedure for unblinding if needed {17b}

The study staff and DSMB may be unblinded to the therapy assignments for regulatory safety reporting of serious adverse events at the request of the DSMB.

## Data collection and management

### Plans for assessment and collection of outcomes {18a}

Labor and delivery medical records will be abstracted to determine the primary outcome. At baseline and 12 weeks, questionnaires detailing tobacco behavior and biomarkers of tobacco use will be collected. Self-reported average cigarettes smoked per day and 7-day point prevalence abstinence from smoking will be assessed at both follow-up time points, and biochemical verification of abstinence at clinic visit 2 will be assessed via end-expired CO and serum cotinine.

### Survey measures

Surveys employed throughout the course of the study cover the following fields and are summarized in Table 1.

**Table 1:** Study assessments

Survey assessments	
Construct	Measures
**Tobacco-related variables**	Fagerstrom Test of Nicotine Dependence (FTND-modified)
	Minnesota Nicotine Withdrawal Scale (MNWS)
	Questionnaire on Smoking Urges (QSU-Brief)
	Smoking Behaviors (Cigarettes per day, quit attempts, quit aides, etc)
**Other substance use**	Alcohol Use Disorders Identification Test (AUDIT-C)
	Marijuana, cocaine, opioids, stimulants, drugs by injection (Veterans Aging Cohort Study-VACS, *modified*)
**Psychological factors**	Patient Health Questionnaire (PHQ-8)
	Generalized Anxiety Disorder (GAD-7)
	Connor-Davidson Resilience Scale (CD-RISC2)

### Biochemical measures

Total lipids will be extracted from 200 μl of double washed packed red blood cells to determine RBC phospholipid membrane fatty acid content using the method described by Folch et al. [44]. The lowest level of detection for individuals’ fatty acids is less than 0.5% of the total profile. We will use the Smokerlyzer ED50 CO to assess end-expired carbon monoxide. Biochemically validated abstinence will be defined as end-expired carbon monoxide less than 4 ppm [1]. Cotinine levels and 3-OH cotinine levels used to calculate the nicotine metabolite ratio will be measured using isotope dilution high-performance liquid chromatography/atmospheric pressure chemical ionization tandem mass spectrometry [1, 45].

### Plans to promote participant retention and complete follow-up {18b}

We will try to improve retention by [1] timing all research-related activities to occur during scheduled clinic visits, [2] limiting the amount and length of a visit (estimated 30–40 min additional time), [3] sending periodic text reminders to our population to keep them engaged in the study and reinforce compliance with the medication, and [4] offering study reimbursement. In our pilot study, increasing study reimbursement rates helped to reduce no-show rates. Higher-than-anticipated rates of loss to follow-up should only impact our secondary outcomes, as tobacco exposure would require an in-person visit. Our primary outcome will be abstracted from medical records, and as such we anticipate more complete records. We will include text reminders, birthday cards and study newsletters, and additional incentives such as small gifts.

### Data management {19}

All study data will be captured electronically and stored via a secure, web-based data capture system, REDcap™ [46]. Paper forms will be used during enrollment, if needed, and stored securely in a locked cabinet that only study staff will have the ability to access.

Quality control measures will include detailed and unambiguous specifications for completion of data forms, including rules for coding skipped questions and missing data, training of study staff responsible for data collection, and built-in validation rules, error checks, question skips for electronic data capture, and computer algorithms to check for out-of-range codes and internal inconsistencies. All data, regardless of capture method, will be reviewed for logic, skip patterns, response ranges, out-of-range codes, and internal inconsistencies, and converted to SAS (or equivalent statistical package) datasets for analysis.

### Confidentiality {27}

To prevent the loss of data, all electronic information will be stored within the VUMC firewall and is password-protected. Hard-copy, original consent forms and any other study documents that include participant names and contact information will be kept in binders and locked in a filing cabinet. Electronic participant data will be placed into a password-protected, web-based database on encrypted computers and iPads by study personnel. Electronic medical records and electronic participant tracking spreadsheets will be stored on a secure server. Only the research staff and the study investigators will have access to this data.

All procedures are in accordance with best practices for Federal Health IT as determined by the Office of the National Coordinator for Health Information Technology. Protected health information will not be exchanged outside of the approved study personnel at Vanderbilt University Medical Center.

### Plans for collection, laboratory evaluation, and storage of biological specimens for genetic or molecular analysis in this trial/future use {33}

If the participant consents to genetic testing and/or future studies, approximately 5 mL of blood will be collected at enrollment and clinic visit 2. Blood samples will be collected at all in person study visits. Blood will be drawn into two 10 ml EDTA tubes and transferred to the trial laboratory the day of collection for processing and storage in − 80 °C freezers. For carbon monoxide determinations, participants will be advised to hold their breath for 15 seconds before blowing in the device. The results of the device will be recorded into the REDCap database. These samples will be securely stored in a laboratory at VUMC until analysis.

## Statistical methods

### Statistical methods for primary and secondary outcomes {20a}

Baseline covariate measures for the two treatment arms will be summarized with means (SDs), medians (IQR), and categorical percentages as appropriate. Standardized differences will be reported to quantify the balance achieved through the randomization. The primary test for statistical significance for continuous and semi-continuous outcomes, which includes the primary outcomes for aims 1 and 2—gestational age at birth and percent change in cigarettes per day—will be an unequal variance two-sample *t* test. Note that while the distribution of both primary outcome measures will have skewness, our preliminary work has shown that a sample size of 400 participants is sufficient for the t-test to perform well. The primary analysis will be intent-to-treat—i.e., subjects will be included in the group to which they were randomized regardless of compliance with treatment. Effect size estimation will be the mean difference between treatment arms in the outcomes, with 95% confidence intervals calculated using the variance and critical value corresponding to the primary hypothesis test, i.e., a t-distribution with degrees of freedom estimated via the Satterthwaite approximation.

For dichotomous outcomes, which include the secondary outcomes fetal death/stillbirth and biochemically validated 7-day point prevalence abstinence from smoking at 12 weeks, statistical significance will be tested using a chi-square test with the Yates continuity correction. Effect size estimation will be the risk difference with 95% confidence intervals calculated using the Agresti-Caffo interval. All outcomes will be reported as being one of the two primary or as a secondary outcome.

The performance of the proposed statistical methods and estimated statistical power were done through simulating batches of 20,000 datasets under various settings. To account for the potential skew in some outcomes, transformations of the Poisson distribution were used to generate outcome data with the placebo arm's mean and standard deviation calibrated to those observed in Coleman, et al. [47]. Detectable effect sizes were calculated at 80% and 90% power. They are presented on the clinical scale for the primary outcomes and presented as standardized effect sizes for generalized estimates applicable to the continuous secondary outcomes. Aim 1 is as follows: a 0.55-week difference in mean gestational age at delivery is detectable at 80% power; a 0.63 week difference at 90%. Aim 2 is as follows: assuming a common standard deviation for percent cigarettes per day reduction of 15%, the study will detect mean differences of 4.3% and 4.9% with 80% and 90% power, respectively. Secondary outcomes are as follows: these detectable effect sizes correspond to standardized effect sizes of 0.29 and 0.33, respectively.

For dichotomous outcomes, all of which are secondary or exploratory, the detectable effect size depends on the outcome prevalence in the placebo arm. Assuming a 10% preterm delivery rate in the placebo arm, risk differences of 7% and 8% are detectable with 80% and 90% power, respectively. Similar risk differences are detectable assuming a 10% smoking cessation rate. These risk differences correspond to relative risks of 0.28 and 0.21.

### Interim analyses {21b}

Interim analyses will be prepared for the DSMB by the study biostatistician. The results will be blinded and presented on a coded group basis (i.e., A or B groups). The committee will have the authority to modify the study protocol or terminate the study if they deem such actions to be warranted. The DSMB will provide these summary reports to the VUMC Institutional Review Board (IRB), the National Institutes for Health (NIH), and the investigators.

### Methods for additional analyses (e.g., subgroup analyses) {20b}

The first subgroup to be examined will be defined by gestational age at birth. If a significant n-3 LCPUFA supplementation—gestational age at birth association—is observed, we will conduct an exploratory analysis to determine what proportion of this association is mediated through smoking reduction and what proportion through an increase in circulating n-3 LCPUFA levels. The ability of this analysis to differentiate between these two possible mechanisms will depend on how strongly correlated the two are. However, provided there are a sufficient number of participants who increase their n-3 LCPUFA without reducing their cigarettes per day, this study will be in a unique position to investigate additional potential mechanisms of n-3 LCPUFA supplementation and reduced risk of preterm birth. Additional analyses are planned among sub-groups as defined by the following: [1] baseline n-3 LCPUFA levels, [2] baseline cigarettes per day, [3] hepatic nicotine metabolism as assessed by the nicotine metabolite ratio [1, 45], and [4] baseline anxiety or depression as assessed by validated questionnaires (Table 1). If a significant interaction is observed between n-3 LCPUFA supplementation and any of these pre-specified subgroups, we will conduct analyses in each stratum of the subgroup to determine the effect size and 95% confidence intervals.

### Methods in analysis to handle protocol non-adherence and any statistical methods to handle missing data {20c}

Unused medications will be collected at clinic visit 2 for pill counts, and participants will be asked to self-report medication compliance. RBC membrane content of n-3 LCPUFA will be measured as a biomarker of compliance [48, 49]. Dropout rate will be compared between treatment arms. Tipping point sensitivity analyses on dropout will be performed to determine what conditions among those missing outcomes would be needed to tip the primary conclusions to statistical non-significance. Women who are missing data at 12 weeks on smoking status will be assumed to be active smokers. Women who are completely lost to follow-up will be considered to have had a normal delivery (gestational age of 38 weeks). We will obtain medical records related to delivery and do not anticipate much missing data regarding gestational age at birth.

### Plans to give access to the full protocol, participant level-data, and statistical code {31c}

The full protocol will be available as a supplemental file with publication of the first manuscript including outcomes data. Requests for participant level-data and statistical code will only be accessible through a formal review of the data request by the study steering committee.

## Oversight and monitoring

### Composition of the coordinating center and trial steering committee {5d}

The organization of the INFANTS study is presented in the below figure. The INFANTS study will include a steering committee which will include representatives from the Department of Obstetrics and Gynecology at the two clinical centers and the study biostatistician. The steering committee will provide overall governance for the trial. The study principal investigators will receive administrative and regulatory guidance from VUMC IRB, the DSMB, and the steering committee. The study PIs will report study progress and milestones to the NIH program officer via annual reports and more often as needed. Data collected at clinical sites will be transmitted to the data coordinating center at VUMC via HIPAA-compliant electronic data capture (REDCap). Study administrative activities such as trial reporting and grant administration will occur at VUMC under the guidance of the steering committee and will be the responsibility of the PIs.

### Composition of the data monitoring committee, its role and reporting structure {21a}

We will form a three-member independent DSMB. The members of the DSMB will not be involved with the project, will have no conflicts of interest, and will be selected so that they provide the appropriate clinical research and safety expertise. The function of the DSMB will be to provide objective, independent review of results, particularly as they relate to patient safety.

### Adverse event reporting and harms {22}

Safety and tolerability information regarding gastrointestinal will be collected systematically thought direct query of the participants while other potential adverse events will be collected through participant self-report and asking about non-specific events. Rates of AEs will be reported based on trial arm in trail publications. During the conduct of the trial, any serious adverse event will be reported to the DSMB within 24 h. Board members will be provided all the available clinical data surrounding the clinical occurrence. During its regularly scheduled meetings, the DSMB will also be provided with a list of non-serious adverse events organized by treatment group. The DSMB will be charged with the prompt review of this information and with providing feedback to the PIs as necessary. The DSMB will have access to the study biostatistician to provide additional data or analysis if required.

### Frequency and plans for auditing trial conduct {23}

Two interim analyses have been planned for specific safety and trial futility analyses. The first interim analyses will occur after 50 participants have been enrolled in the study and completed all research-related interventions (post-delivery). A second interim analysis is planned after 200 participants have completed all research-related interventions. The study statistician will develop the group sequential boundaries which will be in place as study stopping rules. The DSMB will reports its minutes to the VUMC Institutional Review Board.

### Plans for communicating important protocol amendments to relevant parties (e.g., trial participants, ethical committees) {25}

Protocol amendments will be communicated to the VUMC IRB according to standard policies and procedures, and updates to Clinicaltrials.gov will be made accordingly. Should an amendment involve a change in the informed consent documents, subjects will be re-consented.

### Dissemination plans {31a-31b}

Key findings from this research will be presented at national and international conferences and reported in peer-reviewed journals. There is no intended use of professional writers and authorship for future trial publications will be defined using ICMJE definitions with roles defined based on journal criteria.

## Discussion

INFANTS is a multi-center, randomized, placebo-controlled, double-blind study of n-3 LCPUFA supplementation in pregnant smokers. In conjunction with collecting validated biological markers of cigarette exposure (plasma cotinine and end-expiratory carbon monoxide) and biomarkers of n-3 LCPUFA status (i.e., red blood cell phospholipid membrane fatty acids), we will collect detailed self-reported information regarding smoking behaviors. Collectively, this information will be used to determine the effect of supplemental n-3 LCPUFAs on gestational age at delivery (primary study outcome), preterm labor, and tobacco use in pregnant smokers.

## Trial status

Trial enrollment was originally planned to begin in April 2020, but due to the COVID-19 pandemic, it was delayed until November 2020. The study is anticipated to conclude the spring of 2024.

## Abbreviations

Audit-C: Alcohol Use Disorder Identification Test
CD-RISC: Connor-Davidson Resilience Scale
CO: Carbon monoxide
CPD: Cigarettes per day
DSMB: Data and Safety Monitoring Board
FDA: Federal Drug Administration
GAD: Generalized Anxiety Disorder Assessment
GI: Gastrointestinal
EHR: Electronic health record
IDS: Investigational Drug Service
IQR: Interquartile range
IRB: Institutional Review Board
LMP: Last menstrual period
MNWS: Minnesota Nicotine Withdrawal Scale
n-3 LCPUFA: n-3 long-chain polyunsaturated fatty acid
NIH: National Institutes of Health
NRT: Nicotine replacement therapy
OB/GYN: Obstetrician/gynecologist
OTC: Over the counter
PHQ: Patient Health Questionnaire
PPM: Parts per million
PRAMS: Pregnancy Risk Assessment Monitoring System
QSU: Questionnaire on Smoking Urges
RA: Research assistant
RASF: Research assistant screening form
RBC: Red blood cell
RCT: Randomized control trial
SCRIPT: Smoking Cessation and Reduction in Pregnancy Treatment
SDs: Standard deviations
TTS: Tobacco treatment specialist
VUMC: Vanderbilt University Medical Center
